# Hyaluronan modulates TRPV1 channel opening, reducing peripheral nociceptor activity and pain

**DOI:** 10.1038/ncomms9095

**Published:** 2015-08-27

**Authors:** Rebeca Caires, Enoch Luis, Francisco J. Taberner, Gregorio Fernandez-Ballester, Antonio Ferrer-Montiel, Endre A. Balazs, Ana Gomis, Carlos Belmonte, Elvira de la Peña

**Affiliations:** 1 Instituto de Neurociencias, Universidad Miguel Hernández-CSIC, San Juan de Alicante, 03550 Alicante, Spain; 2 Instituto de Biología Molecular y Celular, Universidad Miguel Hernández, Elche, 03202 Alicante, Spain; 3 Matrix Biology Institute, 1040 Arcadian Way, Fort Lee, New Jersey 07024, USA

## Abstract

Hyaluronan (HA) is present in the extracellular matrix of all body tissues, including synovial fluid in joints, in which it behaves as a filter that buffers transmission of mechanical forces to nociceptor nerve endings thereby reducing pain. Using recombinant systems, mouse-cultured dorsal root ganglia (DRG) neurons and *in vivo* experiments, we found that HA also modulates polymodal transient receptor potential vanilloid subtype 1 (TRPV1) channels. HA diminishes heat, pH and capsaicin (CAP) responses, thus reducing the opening probability of the channel by stabilizing its closed state. Accordingly, in DRG neurons, HA decreases TRPV1-mediated impulse firing and channel sensitization by bradykinin. Moreover, subcutaneous HA injection in mice reduces heat and capsaicin nocifensive responses, whereas the intra-articular injection of HA in rats decreases capsaicin joint nociceptor fibres discharge. Collectively, these results indicate that extracellular HA reduces the excitability of the ubiquitous TRPV1 channel, thereby lowering impulse activity in the peripheral nociceptor endings underlying pain.

## Abstract (web summary)

Hyaluronan is a major component of the extracellular matrix, and is used to treat joint pain in osteoarthritis. In this study, Caires *et al.* show hyaluronan achieves its analgesic effects by targeting TRPV1 and stabilising the channel in its closed state.

## Graphical abstract

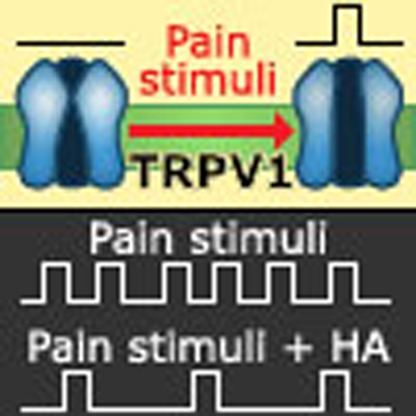

Hyaluronan (HA) is an anionic linear polymer that is ubiquitously expressed in the extracellular matrix (ECM) of mammalian tissues, where it forms loose and elastic matrices^1^,^2^. HA is a space filling molecule that makes the ECM an appropriate environment for cell movement and proliferation and confers elastoviscous biomechanical properties to the tissues^3^,^4^. In addition, HA interacts with specific proteins, such as TSG-6, inter-α-trypsin inhibitor, pentraxin and trombospondin 1, and with membrane receptors, such as CD44, RHAMM, HAHARE and Toll-like receptor 4/2, thereby modulating the development, morphogenesis, cell migration, apoptosis, cell survival, and inflammation and tumorigenesis^5^,^6^. In addition, HA modulates stretch-activated channels^7^, and Ca(v)1.2 channels in neurons^8^.

In joints, HA is continuously secreted by the lining cells of the synovial membranes and provides a protective rheological buffer that reduces the force transmitted by joint movements to joint tissues, including nociceptive nerve endings^9^,^10^,^11^. In chronically injured and/or inflamed arthritic joints, elastoviscosity of the synovial fluid becomes abnormally low because of the dilution and degradation of HA^12^,^13^. The presence of small size HA molecules in the synovial fluid not only alters its rheological properties but also facilitates the production of pro-inflammatory cytokines^14^, thereby contributing to sensitize nociceptive terminals and enhancing spontaneous and movement-evoked joint pain^15^. Notably, intra-articular injection of HA alleviates pain in osteoarthritic patients^16^,^17^,^18^, attenuates lameness in natural and experimentally induced osteoarthritis in horses^19^, and decreases the augmented movement-induced nerve impulse activity in sensitized joint nociceptor fibres^10^,^11^. However, the molecular mechanisms underlying HA anti-nociceptive activity remain poorly understood.

Here, we explored the hypothesis that the analgesic effects of HA in joints are partially mediated through the modulation of TRPV1 channel activity in nociceptive terminals. TRPV1 is a non-selective cationic channel preferentially expressed by primary nociceptive neurons that has been implicated in arthritic pain^20^,^21^,^22^,^23^. Accordingly, the pharmacological modulation of TRPV1 has been shown to produce anti-nociception in arthritis animal models^24^,^25^. We observed that HA inhibits TRPV1 channel activity and reduces action potential firing in nociceptive neurons and that it shows a previously unknown molecular mechanism that explains the attenuation by HA of peripheral nociceptor activity and pain.

## Results

### HA reduces calcium responses to heat and capsaicin

**Figure 1:**
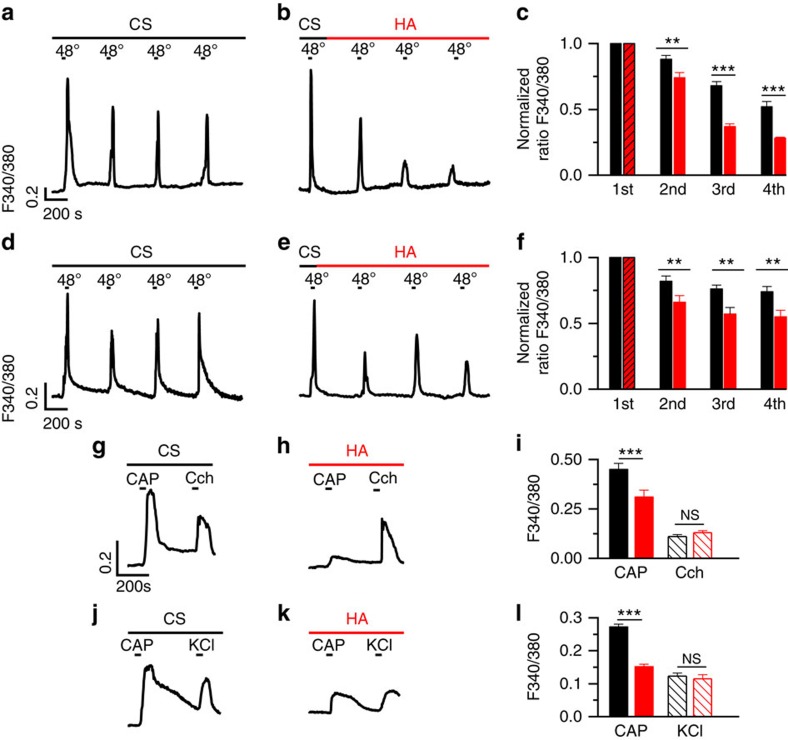
Inhibition by HA of intracellular calcium responses to heat (48 °C) and 100 nM CAP in HEK-TRPV1-EYFP (+) cells and DRG primary sensory neurons. (**a**) Intracellular calcium rises evoked in a HEK-TRPV1-EYFP (+) cell by temperature elevations of the bathing solution to 48 °C repeated at 10 min intervals. Cytosolic Ca^2+^ increases are represented as the ratio of the emission fluorescence intensities at 340 and 380 nm. Notice the development of desensitization. (**b**) The same experiment as in **a** but with a HA perfusion, at the end of the first heating stimulus. (**c**) The normalized ratio of average amplitude change between responses evoked by successive heat pulses (indicated in the abscissae axis) in the control solution (CS, black bars) and during perfusion with HA (red bars). Striped red bar represents the average amplitude of the response in the control saline solution for cells treated with HA immediately afterwards. Notice that the inhibition was maximal at 30 min after the onset of the HA perfusion. (**d**–**f**) The same protocol as in **a**–**c** but performed in cultured adult DRG primary sensory neurons. The inhibitory effect was maximal after 20 min of HA perfusion (third versus first stimuli). (**g**,**h**) Intracellular calcium change in a HEK-TRPV1-EYFP (+) cell in response to 100 nM CAP and to 100 μM carbachol (Cch), a compound that activates endogenous muscarinic receptors in HEK293 cells, applied in CS (**g**) and after exposure to HA initiated 30–60 min earlier (**h**). (**i**) The average amplitude of the response to CAP (filled bars) and Cch (striped bars) under perfusion with CS (black, *n*=68) and in the presence of HA (red, *n*=100). (**j**–**k**) Intracellular calcium responses of DRG adult cultured sensory neurons to 100 nM CAP and to 30 mM KCl during perfusion with the control solution (**j**) and with HA (**k**). (**l**) The average amplitude of the intracellular calcium responses of DRG neurons to CAP (filled bars) or 30 mM KCl (striped bars) in control solution (black) and in the presence of HA (red). Note that the [Ca^2+^]_i_ increase evoked by membrane depolarization of CAP-sensitive neurons with 30 mM KCl was not altered by HA. The data are represented as the mean±s.e.m. Student's *t*-test: ***P<0.001; **P<0.05; ^NS^*P*>0.5.

We analysed the effects of HA on TRPV1-EYFP channels expressed in HEK293 cells and in dissociated DRG primary sensory neurons. We measured changes in intracellular calcium concentration [Ca^2+^]_i_ evoked by brief noxious heat stimuli (48 °C) applied during perfusion with a control solution (CS) or HA (Fig. 1a,b). Repeated heat pulses (P1–P4) under a CS perfusion induced an amplitude decay of the [Ca^2+^]_i_ response (desensitization, 30% at P3 and 50% at P4, *n*=55) that was significantly larger in the presence of HA (60% at P3 and 70% at P4, *n*=74) (Fig. 1a–c) and consistent with the inhibitory activity of HA. Inhibition persisted after a 50-min washout of the cells with the CS (Supplementary Fig. 1).

Using a similar protocol, we further investigated whether HA also inhibited [Ca^2+^]_i_ nociceptive DRG neuron responses to heat in culture (Fig. 1d–f). Desensitization during P3 and P4 in DRG neurons responding to noxious heat during perfusion with the CS was 25% (Fig. 1d,f; *n*=92), whereas under exposure to HA, the amplitude of the response to P3 and P4 was 44% lower on average than P1 (Fig. 1e,f, *n*=34). This result indicates that HA significantly inhibited heat-evoked [Ca^2+^]_i_ rises in nociceptor neurons in culture. In HEK-TRPV1-EYFP (+) cells and DRG neurons, we observed that HA also inhibited CAP-evoked [Ca^2+^]_i_ elevations (Fig. 1g–l). In the CS, 92% (*n*=121) of the TRPV1-EYFP (+) cells responded to 100 nM capsaicin (CAP). By contrast, this percentage decreased to 63% (*n*=175) with exposure to HA; furthermore, the amplitude of the [Ca^2+^]_i_ rise in HA was 31% lower than in the CS (Fig. 1i). Similar results were obtained using a low pH as the TRPV1 stimulus (Supplementary Fig. 2). Similarly, the CAP-evoked [Ca^2+^]_i_ increase in DRG neurons was significantly affected by HA (Fig. 1j,k). In the CS conditions, 68% of DRG neurons (*n*=276) responded to 100 nM CAP, decreasing to 37% (*n*=360) in the presence of HA. In addition, the amplitude of the response to CAP in HA-treated DRG neurons was 44% lower than in those exposed to CS (Fig. 1l). We also used DRG neurons from *TRPV1* null mice as controls. These neurons were unresponsive to capsaicin but remained heat activated. Supplementary Table 1 shows that HA reduced the heat response amplitude in wild-type (WT) sensory neurons by 63% but only by 26% in *TRPV1*^*−/−*^ DRGs. The effect of HA on the heat response was, on average, 2.4 times larger in WT sensory neurons than in *TRPV1*^*−/−*^ neurons. The residual response to heat observed in DRG-*TRPV1*^*−/−*^ sensory neurons was attributable to the activation of other thermosensitive channels. All DRG heat-sensitive neurons from WT animals responded to CAP, as expected, whereas no neurons in *TRPV1*^*−/−*^ mice were activated by the vanilloid, although they responded to 30 mM KCl, thus confirming the absence of TRPV1 channels in *TRPV1*^*−/−*^ mice DRG neurons. A thermal threshold to heat was not modified by HA in TRPV1-EYFP-HEK293 cells or in DRG neurons of WT and *TRPV1*^*−/−*^ individuals, although it decreased the amplitude of the calcium response to 48 °C. HA also reduced the rate of the heat-induced calcium rise in TRPV1-EYFP-HEK293 cells and DRG neurons of WT mice but not *TRPV1* null mice. This result is consistent with the tenet that sensitivity of the TRP channels to temperature appears whenever the activation energies associated with the opening and closing transitions are sufficiently different and not governed by a single characteristic thermal threshold^26^.

Finally, we determined the half maximal inhibitory concentration (IC_50_) of HA using SHSY5Y-TRPV1 cells that express stable levels of TRPV1 channels and measured [Ca^2+^]_i_ increases evoked by 48 °C pulses, under the CS and increasing HA concentrations (50, 200, 400 and 800 μg ml^−1^). The dose–response relationship showed a correlation between HA concentration and its inhibitory effect on the Ca^2+^ response with an IC_50_ of 135±3 μg ml^−1^.

### HA inhibits capsaicin-evoked TRPV1 ionic currents

**Figure 2:**
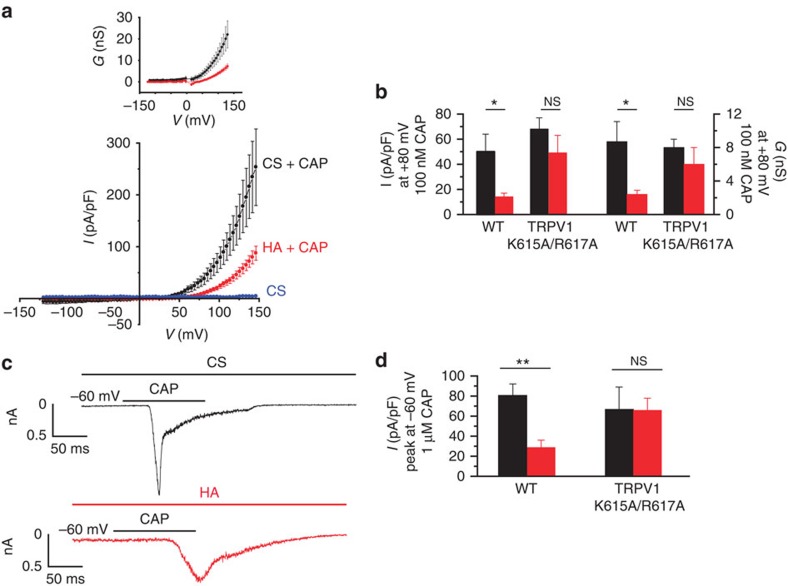
Inhibition by HA of CAP-evoked currents in HEK-TRPV1 cells. (**a**) Average *I*–*V* relationships before (blue symbols, CS) and after the application of 100 nM CAP (black symbols, CS+ CAP; *n*=19) and after the application of HA + 100 nM CAP (red symbols; *n*=18) in HEK-TRPV1-EGFP(+) cells preincubated with HA for 30–60 min. Inset, *G*–*V* curves obtained from the *I*–*V* relationships. The values of different parameters were measured from the *I*–*V* ramps fit with the Boltzmann function *I=gx* (*V*−*E*_R_)/(1+exp ((*V*_1/2_−*V*)/*S*)). In control, *G*_max_*=*45±7 nS, *V*_1/2_=151±12 mV, *z*_g_=0.6, (*n*=19), in the presence of HA: *G*_max_ 22±2 nS, *V*_1/2_ 147±10 mV, *z*_g_=0. 7 (*n*=18). (**b**) The average current (left axis) and average conductance (right axis) at +80 mV obtained from the *I*–*V* relationship shown in **a** in the WT and TRPV1 K615A/R617A mutated channel. (**c**) Whole-cell currents at −60 mV in response to 1 μM CAP in CS (black trace) and cells preincubated with HA for 30–60 min (red trace). (**d**) The average values of peak currents evoked by 1 μM CAP at −60 mV in the WT and TRPV1 K615A/R617A mutated channel, in CS (black, *n*=8) and in the presence of HA (red, *n*=6). The data are represented as the mean±s.e.m., Student's *t*-test: **P<0.01; *P<0.05; ^NS^*P*>0.5.

We performed whole-cell voltage-clamp recordings in HEK-TRPV1-EYFP(+) cells to define the influence of HA on membrane currents induced by the activation of TRPV1 channels. CAP was applied to activate TRPV1 in the absence and presence of HA (Fig. 2a). We calculated the value of the EC_50_ for the CAP response in cells perfused with the CS or pretreated with 400 μg ml^−1^ HA (5.2 MDa) for 30 min before CAP (0.1, 0.25, 0.5, 1 and 10 μM) was instilled. The EC_50_ was similar in both conditions (0.33±0.05 μM in CS and 0.36±0.01 μM in HA) with a Hill coefficient *n*_H_>1 in both cases, thus supporting the tenet that HA does not act as a competitive capsaicin antagonist. TRPV1 current density was reduced by 70% in the presence of HA at +80 mV (Fig. 2b) and by 64% at −60 mV (Fig. 2c,d).

Although HA has a negative charge, *N*-glycosylation of TRPV1 did not affect polymer inhibitory activity (Supplementary Fig. 3). Analysis of the activation slope (Fig. 2c) showed a value of 92±9 pA ms^−1^ in the CS that HA reduced to 15±3 pA ms^−1^, which indicated that HA slowed the channel current activation.

We then analysed the voltage dependency of TRPV1 gating in CS and the presence of HA by fitting the *I*–*V* relationships (Fig. 2a) to a Boltzmann-linear function^27^. HA did not alter the voltage required to activate 50% of the maximal conductance (*V*_1/2_) or the gating valence of channel activation (*z*_g_). By contrast, HA reduced the maximal channel conductance (*g*) by ≈50%. Altogether, these data implied that HA inhibited TRPV1 by affecting the channel's current flow and activation kinetics.

### HA reduces the opening probability of TRPV1 channels

**Figure 3:**
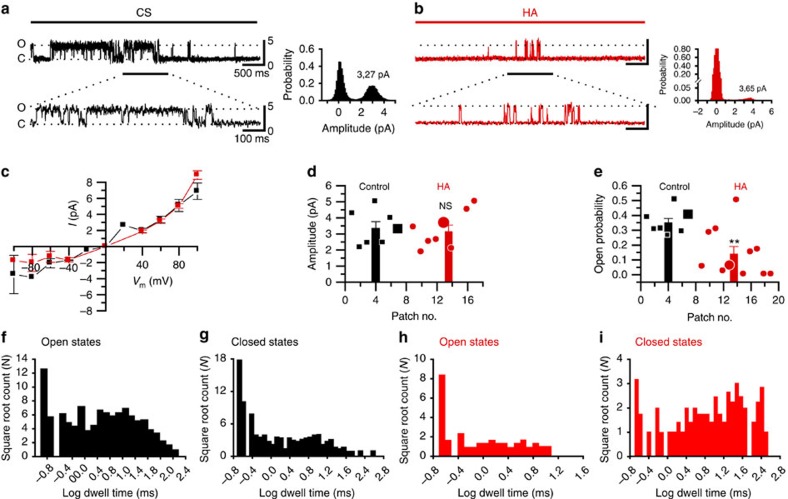
Modulation of TRPV1 single-channel activity by HA. Recording in cell-attached patches of HEK-TRPV1-EFYP (+) cells of the single-channel activity evoked by 0.25 μM CAP in the recording pipette. (**a**) A sample recording of TRPV1 single-channel activity under perfusion with CS. (**b**) A sample recording of single-channel activity from a cell preincubated 30–60 min in HA and recorded under perfusion with HA solution. Insets, single-channel amplitude probability histogram of each recording: black, control; red, in a patch treated with HA. The same scale bar values for **a** apply to **b**. (**c**) *I*–*V* curves obtained in CS (black symbols, *n*=7) and after exposure to HA (red symbols, *n*=8). (**d**) Single-channel amplitudes obtained from individual patches. The bars correspond to the mean values of single-channel amplitudes±s.e.m in each condition. (**e**) The open probability of different patches in CS (*n*=7) in HA (*n*=8). In **d** and **e** larger symbols represent the data from the measures performed in the traces shown in **a** and **b**. The bars represent the mean values of single-channel open probability±s.e.m. (**f**–**i**) The open and closed states dwell time distributions from the traces shown in **a** and **b**. Black, control; red, after treatment with HA. Long closing states that were infrequent in CS but increased their frequency in HA. All data were obtained at +60 mV. The data are represented as the mean±s.e.m., Student's *t*-test: **P<0.01; ^NS^*P*>0.5.

To further obtain a mechanistic insight on the HA inhibitory effects on TRPV1, we recorded TRPV1 single-channel activity in HEK-TRPV1-EYFP(+) cells. TRPV1 channel activity in response to CAP was observed in all control patches (*n*=7); by contrast, this response was absent in 3 of the 11 patches of cells recorded in the presence of HA. The current amplitude histogram in the CS showed two obviously separated current peaks representing the open and closed states (Fig. 3a, black inset). HA reduced the area of the open state (Fig. 3b, red inset), which resulted in a 60% decrease of the open probability (Fig. 3e). By contrast, single-channel *I*–*V* relationships (Fig. 3c,d) indicated that HA did not affect the single-channel conductance.

Single-channel kinetics was further evaluated comparing dwell time histograms for the open and closed channel times in the CS (*n*=5) and HA (*n*=4; Fig. 3f–i). Inhibition of channel activity by HA became apparent from the increase in the number of long duration events in the closed time histograms (Fig. 3g,i). Fitting dwell time histograms to multiexponential density functions (3 to open events and 4 to closed)^28^ indicated a significant and prominent effect of HA on the time constant of the longest closed state and its percent contribution to the total time that increased twofold in the presence of HA without any effect on open states. The values from dwell time fitted to multiexponential functions were CS (*n*=7): in ms *τ*_o1_=0.8±0.2, *τ*_o2_=7±2, *τ*_o3_=41±8; in % *A*_o1_=39±3, *A*_o2_=43±3, *A*_o3_=18±3; in ms *τ*_c1_=0.3±0.1, *τ*_c2_=2.5±0.6, *τ*_c3_=28±10, *τ*_c4_=343±119; in % *A*_c1_*=*55±6, *A*_c2=_20±3, *A*_c3=_14±2, *A*_c4_*=*8±1. HA (*n*=6); in ms *τ*_o1_=0.6±0.3, *τ*_o2_=4±1, *τ*_o3_=36±12, in %; *A*_o1_=36±7, *A*_o2_=41±3, *A*_o3_=23±4; in ms *τ*_c1_=0.8±0.2*, *τ*_c2_=7±1**, *τ*_c3_=76±20*, *τ*_c4_*=*1023±188*; in % *A*_c1_=35±2**, *A*_c2=_28±2*, *A*_c3=_2 3±2*, *A*_c4=_14±2*. No differences could be observed in the incidence of open states between the conditions (Student's *t*-test: **P<0.01; *P<0.05; ^NS^*P*>0.5).

Collectively, these data suggest that HA inhibited TRPV1 activity by stabilizing the channel in the closed state, thus reducing the open probability and lengthening the macroscopic activation rate.

### HA reduces capsaicin-evoked impulse firing in DRG neurons

**Figure 4:**
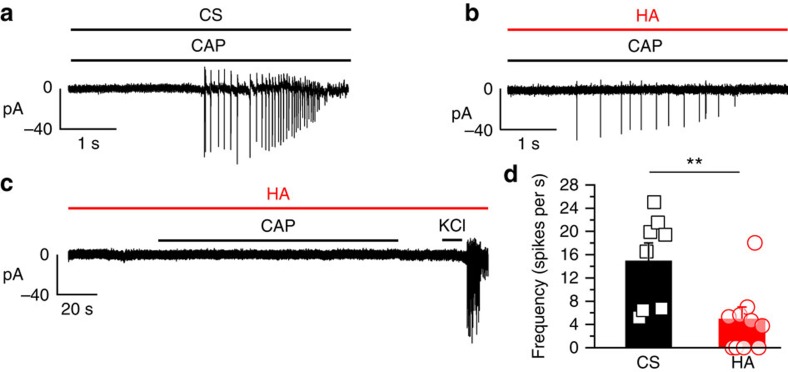
Reduction of the impulse response of DRG neurons to CAP during HA exposure. Electrophysiological recordings of adult cultured DRG neurons performed in the cell-attached configuration during the application of 1 μM CAP, HP*=*−60 mV. (**a**) A sample record of the response to CAP in a single DRG neuron perfused with the control solution with a mean firing frequency of the response: 16 spikes per s (**b**) Sample record of CAP stimulation in a DRG neuron treated with HA and recorded under HA with a mean frequency of the response: 5 spikes per s. (**c**) A sample record of a DRG neuron treated and recorded in HA, in which no response to CAP was observed but an impulse discharge could be evoked with 60 mM KCl. (**d**) The mean firing frequency (columns) and individual data (symbols) of DRGs under CS, black (*n*=8), and after exposure to HA, red (*n*=10). Neurons that did not fire in response to KCl were excluded. Data are represented as the mean±s.e.m., Student's *t*-test: **P<0.01.

The observation that HA inhibits TRPV1 channels prompted us to investigate whether HA also influenced CAP-evoked action potential firing, which we recorded using the cell-attached mode in DRG neurons previously identified as TRPV1 positive by their [Ca^2+^]_i_ response to heat (48 °C). All TRPV1-expressing neurons (*n*=8) perfused with CS fired action potentials when treated with 1 μM CAP (Fig. 4a). The firing frequency of this CAP impulse discharge was reduced in DRG neurons exposed to HA (Fig. 4b). Four out of 10 heat-sensitive neurons did not fire action potentials in response to CAP despite their intact excitability, shown by the preserved responsiveness to 60 mM KCl (Fig. 4c,d). Therefore, these data demonstrated that the inhibition of TRPV1 activity by HA attenuated receptor-induced impulse firing in nociceptive neurons.

### HA prevents TRPV1 sensitization by bradykinin

The sensitization of TRPV1 channels is a critical step in chronic pain^29^. Repetitive, short applications of 100 nM CAP to DRG neurons reached a stable amplitude of the [Ca^2+^]_i_ at approximately the 5th or 6th stimuli (Supplementary Fig. 4a,e). The application of 2 μM bradykinin 2 min before the 6th CAP pulse resulted in a significant increment of [Ca^2+^]_i_, showing TRPV1 potentiation by bradykinin^23^,^30^ (Supplementary Fig. 4b,e,g). Notably, neuron exposure to HA inhibited such TRPV1 potentiation (Supplementary Fig. 4c), thus reflecting a decrease in bradykinin-induced channel sensitization (Supplementary Fig. 4d,f,g). These results confirm a direct action of HA on TRPV1 channels of sensitized peripheral sensory terminals.

### HA reduced behavioural nocifensive responses to noxious heat

**Figure 5:**
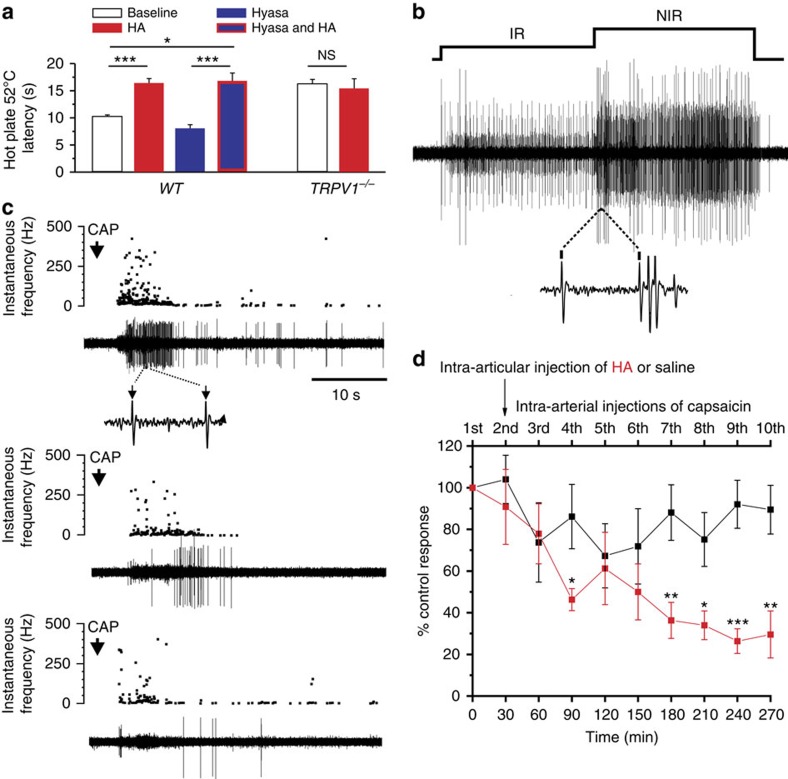
Effect of HA on behavioural nocifensive responses to noxious heat in mice and on nerve impulse activity of sensory nerve fibres innervating the rat knee joint. (**a**) The latency of the first nocifensive response (licking, biting, lifting, guarding, shaking or jumping) obtained in wild-type or *TRPV1*^*−/−*^ null mice in control conditions (baseline) or after receiving a 10 μl injection of HA or Hyasa in the left paw or after receiving an injection of Hyasa followed by another injection of HA in WT (*n*=25,12,11,7) and *TRPV1*^*−/−*^ (*n=*25,18). (**b**) Recording of the multiunit nerve impulse activity in a filament of the saphenous nerve, evoked by an inward rotation of the knee joint in the non-noxious (IR) and the noxious range (NIR) (10 s each). The dotted lines indicate an expanded recording during the marked time interval in which units of different amplitudes have been selected with an amplitude filter. (**c**) Nerve impulse activity evoked by the intra-arterial injection of 100 μl of 10 μM CAP (arrows) performed at 30 min intervals, in the same multiunit filament shown in **b**. In each panel, the instantaneous frequency is represented in the top and original nerve impulse recording, below. Separate units were identified by their amplitude and shape, CAP-evoked discharge started a few seconds after the onset of injection and lasted, on average, 22±2 s (*n*=7). The upper panel corresponds to the control CAP injection and includes an expanded view of the impulse firing during the time indicated between the dotted lines. Notice that this unit evoked by CAP has a similar spike morphology as one of the units recruited by joint rotation in **b**. Middle and lower panels depict the nerve impulse discharge evoked by intra-arterial injection of 10 μM CAP 1 and 2.5 h, respectively, after intra-articular injection of 100 μl of 1% HA. (**d**) The average values of the total number of CAP-evoked impulses over the period of time shown expressed as a percentage of the mean number of impulses evoked by the first (control) intra-arterial injection of CAP (100%). The arrow indicates the time at which either saline (black symbols, *n*=7) or HA (red symbols, *n*=8) were injected intra-articularly into knee joints. The data are represented as the mean±s.e.m., Student's *t*-test: ***P<0.001; **P<0.01; *P<0.05; ^NS^*P*>0.5.

To determine the effect of HA on nociceptive nerve terminals *in vivo*, we tested the latency of the pain response of mice to the hot plate test (52 °C) after subcutaneous injection in the paw of different solutions. In animals injected with saline solution, latency was significantly lower than in animals receiving HA (Fig. 5a). The increment in latency time promoted by HA was not observed in *TRPV1* null mice (Fig. 5a). Similar results were obtained with subcutaneous CAP injection in the hind paw (Supplementary Fig. 5). Hyaluronidase (Hyasa), an enzyme that digests HA of the ECM around pain nerve terminals, caused a significant reduction in latency. Notably, the injection of HA into animals previously treated with Hyasa restored latency values to normal levels (Fig. 5a). Altogether, these results supported the hypothesis that HA reduces the sensitivity of nociceptor endings to noxious stimuli transduced by TRPV1 channels.

### HA inhibits CAP-evoked activity in joint nociceptor fibres

Nociceptor nerve fibres innervating the knee joint synovial membrane, express TRPV1 channels^31^. To explore the possibility that the modulation of nerve impulse activity by HA of the synovial fluid involves TRPV1 channels, we recorded nerve impulse activity in saphenous nerve filaments of anaesthetized rats, selecting polymodal units that responded to noxious rotations of the joint and also to a close intra-arterial, bolus injection of CAP (Fig. 5b,c). In a thin nerve filament, CAP typically recruited 1–6 separate units, responding to CAP with an irregular nerve impulse discharge that started a few seconds after the onset of injection (Fig. 5c). In control experiments, in which intra-articular sterile saline solution was injected immediately after the first intra-arterial CAP injection, the number of impulses evoked by successive CAP injections decayed by a maximum of ≤20% of the impulse discharge produced by the first CAP injection, taken as 100% (control response, black symbols, Fig. 5d). By contrast, when HA was injected intra-articularly, the discharge evoked by serial CAP injections decayed gradually and was ≥60% (Fig. 5c,d). Collectively, these results confirmed that intra-articular HA significantly decreased TRPV1-mediated responsiveness of joint nociceptor fibres.

### HA does not modify TRPA1 or TRPM8 channel activity

To explore the selectivity of HA inhibition on TRPV1 channels, we investigated its effects on TRPA1 and TRPM8, two thermosensory, polymodal TRP channels also expressed by primary sensory neurons^32^,^33^. For TRPA1 channels, we measured [Ca^2+^]_i_ in recombinant systems and mouse-cultured DRG and nodose neurons perfused with CS or HA using nifedipine or cold (35–10 °C) to activate TRPA1 (ref. 34). HA did not affect TRPA1 activity (Supplementary Fig. 6 and Supplementary Table 2). In nodose sensory neurons, HA reduced by 30% CAP-evoked [Ca^2+^]_i_ elevations (Supplementary Fig. 6d) presumably mediated by TRPV1 without affecting responses to noxious cold (10 °C) or nifedipine (Supplementary Fig. 6c). Similarly, HA did not affect TRPM8 responses evoked with cold (20 °C), menthol, or cold combined with menthol application (Supplementary Fig. 6e,f). Thus, these data demonstrate that HA does not modulate TRPA1 or TRPM8 channels.

### A putative HA-interacting site in TRPV1 channels

To biochemically isolate the complex between HA and TRPV1, we used biotin-conjugated HA and anti-TRPV1 to pull-down the (biotin)HA–TRPV1 complex. Notably, although with the obvious evidence of a functional interaction between both molecules, we could not isolate the complex, plausibly because it dissociates while washing non-specific binding. Alternatively, we used molecular modelling as a strategy to learn about the interaction between HA and TRPV1. HA binds to several extracellular membrane proteins through various hyaluronan-binding domains^35^ and short linear sequences containing basic amino acids termed BX_7_B motifs^36^. To model the interaction of TRPV1 with hyaluronate, a global docking process between the extracellular loops of TRPV1, and a HA tetrasaccharide molecule was performed. A tetrasaccharide sufficed to show HA-receptor interactions, as evidenced by the crystal structure of the complex between HA and the CD44 hyaluronan-binding protein^37^.

**Figure 6:**
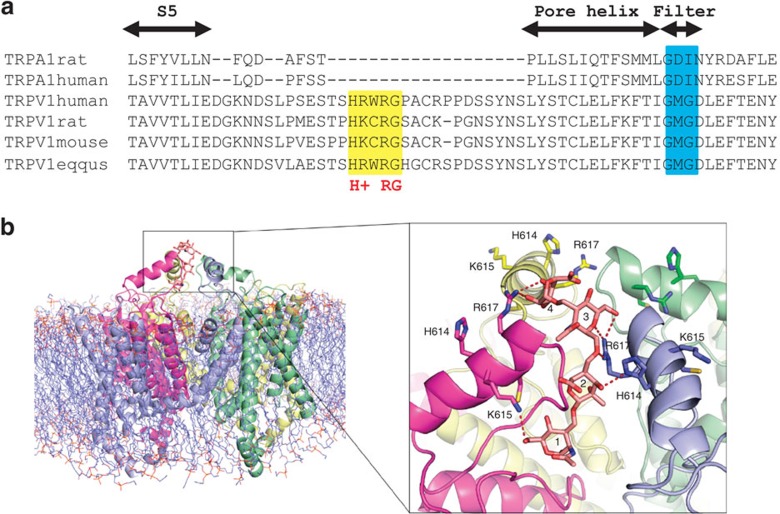
Atomic interactions of hyaluronan tetrasaccharide docked to TRPV1 model. (**a**) Details of the TRPV1 multiple sequence alignment of four species, showing the conservation of the positive patch predicted to interact with HA. Additionally, detail of the multiple sequence alignment between TRPV1 and TRPA1 in different species. A large deletion in TRPA1 S5-pore helix loop losing positively charges could be observed. (**b**) TRPV1 channel model inserted in a lipid bilayer. The proteins are drawn as a ribbon and coloured differently for each subunit. The cytosolic C and N termini have been removed for simplicity. The black square delimits the extracellular loops of TRPV1 and the docked HA. Hyaluronan tetrasaccharide is drawn as sticks and coloured in pastel pink. The numbers indicate the sugar rings, starting from the non-reducing end. The loop side chains involved in the interaction are shown as sticks and coloured accordingly to the colour of its subunit. The dotted lines in red denote hydrogen bonds between atoms closer than 3.2 Å.

The preferred location for putative HA binding in TRPV1 is a patch of positively charged amino acids (sequence 614-HKCRG-618 in rat, consensus [H(K/R)XRG]), located in the extracellular S5-pore helix loop and thus exposed to the solvent (Fig. 6a). Residues involved in the interaction, His614, Lys615 and Arg617, make prominent H-bond contacts with HA (Fig. 6b). In addition, interaction with Lys615 and Arg617 from the contiguous subunit, complete the contact map of HA (Fig. 6b). This putative binding site is consistent with HA receptor selectivity because TRPA1 does not have a positively charged signature in the extracellular domain (Fig. 6a) and thus shows a lower surface electrostatic potential in the external loops and poorer interaction with HA (Supplementary Fig. 7). Furthermore, mutation of K615 and R617 to Alanine (TRPV1 K615A/R617A) produced a mutant channel whose current density was not significantly reduced by HA (Fig. 2b,d). Moreover, the activation slope of this mutant was not affected by HA (42±22 pA ms^−1^, *n*=8 and 24±9 pA ms^−1^, *n*=10, respectively). Therefore, although we could not isolate the HA–TRPV1 complex, our mutagenesis strategy indicated the presence of an HA-binding site in the extracellular channel domain, which was consistent with the major effect of HA modulating the channel open probability and the activation kinetics.

## Discussion

The salient contribution of this study is the demonstration that TRPV1 channels are molecular targets of HA. We provide evidence that in the presence of HA, TRPV1 opens less frequently, thereby decreasing the excitability of peripheral nociceptive neurons and reducing their responsiveness to noxious stimuli. Notably, HA selectively modulated TRPV1 channel function, whereas the activity of related TRP channels, also associated with sensory transduction of noxious and thermal stimuli such as TRPA1 and TRPM8, was not consistently affected by HA.

The inhibitory effect of HA on TRPV1 channels was shown using [Ca^2+^]_i_ increases evoked by physical (heating to 48 °C) and chemical (CAP, low pH) stimulation of TRPV1. However, it should be noted that intracellular Ca^2+^ increased in sensory neurons reflect the overall inflow of calcium through membrane channels, namely TRPV1, directly opened by the stimuli but also Ca_v_ channels activated by TRPV1-induced membrane depolarization. We concluded that the HA inhibition of heat, low pH and CAP-evoked Ca^2+^ influx in sensory neurons appears to be mediated by an action on TRPV1 channels, rather than Ca_v_ channels: (i) HA did not block the Ca^2+^ influx induced by membrane depolarization with 60 mM KCl; (ii) reduction of [Ca^2+^]_i_ increase by HA was also observed when TRPV1 channels were expressed in HEK293 cells; (iii) electrophysiological measurements of TRPV1 channel activity showed that HA reduced the current flow by decreasing the open probability of channel gating; and (iv) the mutagenesis of the putative HA-binding site abrogated HA-mediated TRPV1 antagonism. We further showed that the HA inhibitory activity is independent of the stimulating conditions because HA blocked TRPV1 channels both when they are partially inactivated by repeated noxious stimulation or after being sensitized by bradykinin.

The most likely mechanism underlying HA inhibitory effects is that HA binds to the TRPV1 channel protein and modulates its gating. Our results indicate that HA inhibition of TRPV1 is mediated by a stabilization of the channel closed state. A similar blockade mechanism has been proposed for the inhibitory activity of a polyclonal antibody that binds to the pre-pore loop of TRPV1 (ref. 38). Eleven antibodies, targeting the pore domain of different channels have been identified^39^; our *in silico* docking analysis showed a high probability for an electrostatic-type interaction between HA and a short positively charged sequence ‘H+xRG' located in the external pore domain. This sequence overlaps with the receptor region that holds the binding epitope in TRPV1 of polyclonal functional antibodies^38^. This concurrence supports a common mechanism for HA and polyclonal antibody blockade of TRPV1 channels and identifies a pivotal site for TRPV1 channel modulation. Altogether, our functional results, along with the *in silico* analysis, suggest a direct HA–TRPV1 interaction that downregulates channel activity.

The question arises on how HA could modulate TRPV1 gating. A reasonable molecular explanation according to the most recent structural model of TRPV1 (ref. 40) is that the polymer, by immobilizing the outer pore loop, obstructs this conformational change preventing the associated movement of the pore helix necessary to open the external gate, thereby locking the channel in the closed state. It is also plausible that HA additionally bridges distinct regions on the extracellular domains (S5-P-S6 loop) of the adjacent subunits and even between near TRPV1 channels, clustering the channels and building a complex net or a macromolecular aggregate on the membrane surface. Noteworthy, clustering of TRPV1 receptors in the cell surface inhibits channel activity^41^.

TRPV1 is widely distributed in peripheral and central nervous system neurons and in non-neural tissues^42^,^43^ and has been implicated in a variety of functions, including thermo- and osmoregulation, memory processes or smooth muscle contraction^44^,^45^. Still, the best established role for TRPV1 is to be a molecular detector of thermal and chemical stimuli that activate the sensory neurons to produce acute or persistent pain and inflammation^29^.

Pain in osteoarthritis, the leading cause of physical disability in industrialized nations^46^ is the main symptom in the disease and appears to be associated with TRPV1 expression in humans^47^ and animal models of chronic osteoarthritis^22^,^48^,^49^,^50^. Intra-articular injection of HA decreases joint nociceptor activity in animals and reduces osteoarthritis pain in humans^9^,^16^,^17^,^18^. The current explanation for HA effects on joint nociception is that in intact joints synovial fluid HA acts as an elastoviscous filter for mechanical forces and selectively reduced the transmission during movement of potentially injurious forces to joint structures and nociceptor nerve terminals^51^. When healthy high molecular weight HA (HMW-HA) is broken into its smaller size HA, this elastoviscous filtering capacity decreases. Accordingly, HMW-HA solutions reduce the opening probability of stretch-activated channels in oocytes *in vitro*^7^ and cause a pronounced reduction of movement-induced nociceptor activity when injected intra-articularly in experimentally injured and inflamed knee joints of rats and guinea pigs^10^,^11^. Our results show that synovial fluid HA seems to play additional roles in the modulation of joint nociceptor fibres activity in addition to mechanical filtering. Our results show that healthy HA antagonizes TRPV1 activity and significantly decreases nociceptor excitability also in channels sensitized by the pro-algesic agent bradykinin, thus adding an additional mechanistic explanation to the anti-nociceptive effects of intra-articular HA injections and the possibility of new therapeutic uses of HA. Finally, TRPV1 expression also occurs in other joint cell types, as chondrocytes, osteoclasts, osteoblasts and synovial fibroblasts^42^. Hence, synovial HA, through its inhibitory action on TRPV1 channels in these cells, may also modulate other important biological processes (degeneration, healing) triggered by joint injury and inflammation^52^.

There is emerging evidence that ECM molecules surrounding neurons, which include HA as a major biochemical component, regulate synaptic plasticity in the adult brain^8^,^53^,^54^. TRPV1 has been recently identified in neurons and glial cells of various areas of the central nervous system and is associated with a wide array of functions and behaviours^55^,^56^. For instance, TRPV1 channels in hippocampal neurons contribute to modulate neuronal excitability and their stimulation with CAP enhances 4-AP-induced epileptiform activity *in vitro* and triggers bursting, seizure-like activity *in vivo*^57^. Furthermore, it has been proposed that TRPV1 plays a regulatory role on cortical excitability as well^58^. Hence, it is tempting to speculate that HA of the perineuronal ECM modulates TRPV1 channel activity of hippocampal cells thereby contributing to the control of brain excitability.

## Methods

### Animals

The studies were performed in neonatal and adult C57BL/6JOlaHsd male mice and in adult (1–3 months) *TRPV1*^−/−^ mice (Jackson Laboratory) and Wistar male adult (3–4 months) rats. All experimental procedures were performed according to the Spanish Royal Decree 1201/2005 and the European Community Council directive 2010/63/EU. The Ethics Committee from Universidad Miguel Hernández, Alicante, Spain, approved this study.

### Culture of cells and neurons

The experiments were performed on primary cultures of TRPV1-, TRPM8-, and TRPA1-expressing DRG or Nodose neurons. Previously described culture methods were used for DRG neurons from P1 to P4 and adult mice (1 month)^59^ and nodose ganglion neurons from adult mice (1 month)^34^. The HEK293 cell line (Sigma-Aldrich) transfected with TRPV1+ EYFP fusion protein^60^, N604T-TRPV1-EYFP, K615A/R617A-TRPV1 or hTRPA1 cloned in pAGGS-IRES-GFP (Karel Talavera, Department of Cellular and Molecular Medicine, KU Leuven, Belgium) using Lipofectamine 2000 (Invitrogen) or the CHO-TRPA1 stable cell line (Ardem Patapoutian, The Scripps Research Institute, USA) or HEK293-mTRPM8-YFP cell line (Felix Viana, Instituto de Neurociencias, Alicante, Spain) were used. In some experiments, we used the human neuroblastoma cell line SHSY5Y stably expressing TRPV1 kindly provided by J. Lilja and A. Forsby (Department of Neurochemistry, Stockholm University, Sweden).

TRPV1 mutagenesis: mutations were introduced in the rat TRPV1 construct (from D. Julius) using the Site Directed Mutagenesis (Quick Change II, Agilent Technologies, Santa Clara, CA, USA) according to the manufacturer's instructions. Mutants were confirmed by DNA sequencing.

### Fluorimetric Ca^2+^ measurements

The intracellular Ca^2+^ measurements were performed in single cells loaded with FURA-2AM (Life Technologies, Carlsbad, CA, USA) for 45 min at 37 °C in a 5% CO_2_ incubator. The recordings were performed in a low-volume chamber with a complete solution exchange. Bath control solution contained (in mM) 140 NaCl, 3 KCl, 2.4 CaCl_2_, 1.3 MgCl_2_, 10 HEPES and 10 Glucose adjusted to pH 7.4 with Na(OH). Fluorescence measurements were conducted with a Nikon (Nikon Eclipse TE2000-U) inverted microscope. FURA-2 was excited at 340 and 380 nm with a high-speed monochromator (TILL Photonics, Germany), and the emitted fluorescence was long-pass filtered at 510 nm. The images were acquired using an Andor camera (Oxford Instruments, UK). Acquisition and analysis were performed with TILL vision software (TILL Photonics GmbH, Germany). Cytosolic Ca^2+^ increases are presented as the ratio of the emission intensities of 340 and 380 nm (*F*_340_/_380_: fluorescence arbitrary units). The DRG and nodose ganglion neurons were measured at 37 °C. The cell lines were measured at 20–22 °C, unless otherwise indicated.

### Temperature stimulation

The coverslip pieces with cultured cells were placed in a micro-chamber and continuously perfused (0.5 ml min^−1^) with solutions warmed at 35±1 °C for neurons and 21±1 °C for cell lines. Temperature stimulation was adjusted with a water-heated peltier device placed at the inlet of the chamber and controlled by a feedback device (Warner instruments, Hamden, USA). Heat sensitivity was investigated with fast increase of temperature to 48 °C (ramp of temperature 5 s per °C).

### Electrophysiology in cultured cells

For whole-cell recordings, the bath solution contained (in mM): 140 NaCl, 3 KCl, 2.4 CaCl_2_, 1.3 MgCl_2_, 10 HEPES and 10 glucose adjusted to pH 7.4 with Na(OH) and a pipette solution containing (in mM) CsCl, 5 EGTA and 10 HEPES, adjusted to pH 7.2 with Cs(OH). DRG action currents were recorded using a pipette solution with a concentration of (in mM) 140 KCl, 10 NaCl, 4 Mg-ATP, 0.4 Na-GTP, 10 Hepes pH 7.2 adjusted with KOH, external solution contained (in mM) 140 NaCl, 3 KCl, 2.4 CaCl_2_, 1.3 MgCl_2_, 10 glucose and 10 HEPES, adjusted to pH 7.4 with Na(OH). For single-channel cell-attached configuration, recordings were performed with a bath solution containing (in mM): 140 K-gluconate, 2.5 KCl, 1 MgCl_2_, 5 HEPES, 1.5 EGTA, adjusted to pH 7.4 with K(OH), the pipette solution contained (in mM) Na-gluconate 140, NaCl 10, MgCl2 1, 5 HEPES, 1.5 EGTA adjusted pH 7.2 with Na (OH). Measurements in neurons were performed at 37 °C. Measurements in the cell lines were performed at 20–22 °C, unless otherwise indicated.

Membrane currents in whole cell and action currents in cell-attached configurations were recorded using 5–8 MΩ borosilicate glass capillary patch pipettes. Current signals were recorded with a Multiclamp 700B amplifier and voltage clamp commands were applied using pCLAMP software and a Digidata 1322A digitizer (Molecular Devices, Sunnyvale, CA, USA).

Single-channel, cell-attached recordings were obtained using 10–15 MΩ borosilicate glass capillary patch pipettes in cell-attached configuration maintaining the signalling pathways. The cells were fixed at 0 mV with high-K^+^ extracellular solution. Capsaicin was applied via the patch pipette at 0.25 μM concentration to prevent activation of channels elsewhere in the cell, which may have increased the noise of recording^28^. Current signals were recorded using Axopatch 2B (Molecular Devices, Sunnyvale, CA, USA) patch-clamp amplifier and voltage clamp commands were applied using pCLAMP10 software and a Digidata 1400A digitizer (Molecular Devices, Sunnyvale, CA, USA). Data were collected with the filter set at 2 kHz and sampling frequency 50 kHz.

### Electrophysiological data analysis

Electrophysiological analysis were performed using: pCLAMP10 (Molecular Devices, Sunnyvale, CA, USA), WinASCD software (G. Droogmans, Katholieke Universiteit Leuven, Belgium), and Origin 7.5, OriginLab Corp., Northampton MA, USA). In whole-cell configuration, to estimate whether there was a shift in the voltage dependence of activation of TRPV1 in HEK 293 cells, the current–voltage (*I*–*V*) relationships obtained from repetitive (0.2 Hz) voltage ramps from −120 to +150 mV with a duration of 350 ms applied every 3 s were fitted with a function that combines a linear conductance multiplied by a Boltzmann activation term^27^:


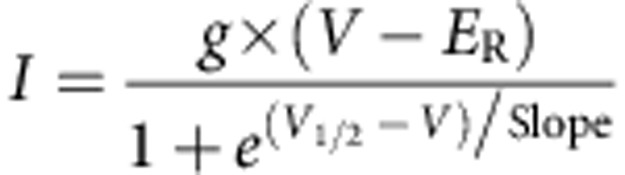


where *g* is the whole-cell conductance, *E*_R_ is the reversal potential of the current, *V*_1/2_ is the potential for half-maximal activation and Slope is the slope factor, which related to the effective gating valence *z*_g_ of channel gating by *z*_g_=25.60 mV per slope.

Single-channel data analysis was performed on patches that contain 1–2 channels. The recordings were analysed with the pCLAMP10.2 Clampfit (Molecular Devices, Sunnyvale, CA, USA). The mean open probability (oP) was measured as from the average *n*oP, where *n* is the number of channels in the patch.

To determinate the distribution of channel open and closed times, dwell time histograms were created, where the square root of number of events *N* was plotted against the dwell time binned on a logarithmic time scale (10 bins per decade).

Dwell time distributions of the open and closed times were fitted to multiexponential density function of the form:


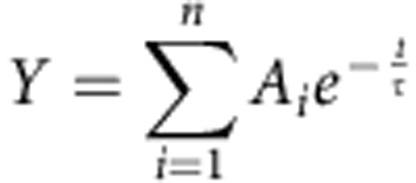


where, *τ*_*i*_ is the time constant of the *i*-th equilibrium and *A*_*i*_ is its amplitude.

All data were expressed as the means (±s.e.m.). Statistical tests included a one-way analysis of variance, the *Z*-test for comparing proportions and Student's *t-* test, as indicated. Differences were regarded as statistically significant with ^NS^*P*>0.05, *P<0.05, **P<0.01 and ***P<0.001.

### Behavioural nociception assays

Adult (3 months) male mice were housed with a 12-h light/dark cycle at 21 °C with food and water *ad libitum*. The mice were habituated to the test environment for 24 h in Plexiglas chambers before the nociception assays and the testing chambers for at least 1 h before testing. The same investigator performed the scoring in all of the behavioural tests, which were blind with respect to the different solutions injected.

We performed intra-plantar injections (into the hind pad of both paws) of hyaluronidase (Sigma-Aldrich) dissolved in physiological control saline (Hyasa (6 U)) or 10 μl of HA solution of high molecular weight HA (average MW=5.2 MDa) at 400 μg ml^−1^ using a 30-G needle. A separate group of animals received a vehicle injection alone. To measure sensitivity to noxious heat, the hot plate test was performed. The temperature of the hot plate was maintained at 52±0.5 °C by a feedback-controlled Peltier device (LE7406Hot Plate, Harvard Apparatus). The latency time to the onset of one of the following nocifensive behaviours was measured: licking, biting, lifting, guarding or shaking of the hind paws, or jumping. Once a score was determined, the heating was switched off. A cut-off time of 30 s was established to avoid tissue injury. The hot plate test was performed every 4, 12, 24 and 72 h and 7–14 days after injection. Capsaicin (1 μg per 10 μl) was injected intradermally into the hind pad, and the duration and score of nocifensive behavior was recorded. In a separate group of mice, these previously (48 h) received an injection of 10 μl HA (average molecular weight=5.2 MDa).

### Electrophysiological recordings in rat knee joint fibres

The experiments were performed in 16 Wistar adult (3–4 months) male rats (mean body weight: 366±10 g) as previously described^61^. Briefly, the animals were initially anaesthetized with ketamine (75 mg kg^−1^) and xylazine (10 mg kg^−1^) (i.p.) followed by an injection of 40 mg kg^−1^ (i.p.) of sodium pentobarbital for deep anaesthesia. Supplementary doses of anaesthetic were injected through a venous catheter when required. The trachea, the left femoral vein and femoral artery were cannulated. Body temperature and CO_2_ were maintained at physiological levels. Heart frequency and blood pressure values were continuously monitored to evaluate the anaesthesia level. An additional catheter was inserted into the right saphenous artery for close intra-arterial injection of substances into the joint area. The right femur was fixed by a special grip, and a pool was formed by skin flaps and filled with warm paraffin oil. The saphenous nerve from the right leg was dissected, and fine filaments were sub-dissected from the peripheral end and placed over a silver wire electrode for extracellular recording.

### Molecular modelling

A TRPV1 model derived from electron microscopy^61^ was used as the starting point to model the absent external loops (604–626) located between the TM S5 and pore helix. TRPA1 was modelled by homology using the spatial coordinates of TRPV1 homology model. The homology models were performed in the Swiss-Model Protein Modelling Server^62^ at ExPASy Molecular Biology site (http://kr.expasy.org/). The assembly of the channels and construction of the missing external loops were performed using DeepView v4.1 (ref. 63), and Yasara^64^ (http://www.yasara.org). The side chains were optimized in two steps: first, residues with van der Waals clashes were selected and fitted with ‘Quick and Dirty' algorithms (DeepView); second, the model was energy minimized. This process involved an initial short steepest descent minimization to remove bumps and a simulated annealing minimization^65^. The model was evaluated using PROCHECK to show residues in the allowed regions of the Ramachandran plots^66^. In addition, the model was tested in terms of energy with FoldX^67^ at the CRG site (http://foldx.crg.es). The force field of FoldX evaluated the properties of the structure, such as its atomic contact map, the accessibility of the atoms and residues, the backbone dihedral angles, and the hydrogen bond and electrostatic networks of the protein.

TRPV1– and TRPA1–carbohydrate complexes were built using the homology models and the hyaluronan molecule (CID 24847767) obtained from the PubChem Compounds database (http://www.ncbi.nlm.nih.gov/pccompound/). A global docking procedure was accomplished with AutoDock 4 (Morris, 2008) implemented in Yasara, where a total of 500 flexible docking runs were established and clustered around the putative binding sites. Using the implemented AMBER 99 force field, the programme then performed a simulated annealing minimization of the complexes that moved the structure to a nearby stable energy minimum^68^. Binding energy was obtained by calculating the energy at an infinite distance between the ligand and channel tetramer and subtracting the energy of the entire complex. The more positive the binding energy, the more favourable the interaction was in the context of the force field. The best binding energy complex in each cluster was stored, analysed and used to select the best orientation of the interacting partners. Figures were drawn with Pymol v1.6 (DeLano Scientific, Palo Alto, CA, USA; http://www.pymol.org).

### Reagents

Sodium hyaluronate solution 400 μg ml^−1^, high molecular weight (≈5.2 MDa; Matrix Biology Institute, Edgewater, NJ, USA), Hyaluronidase, capsaicin, bradykinin, cinnamaldehyde, nifedipine, mustard oil and carbachol were purchased from Sigma-Aldrich (St Louis, MO, USA), and menthol from Sharlau (Spain).

Data are reported as the mean±standard error of the mean (s.e.m). Statistical significance ***P<0.001, **P<0.01, *P<0.05 was assessed by Student's *t*-test.

## Additional information

**How to cite this article:** Caires, R. *et al.* Hyaluronan modulates TRPV1 channel opening, reducing peripheral nociceptor activity and pain. *Nat. Commun.* 6:8095 doi: 10.1038/ncomms9095 (2015).

## Supplementary Material

Supplementary Information Supplementary Figures 1-7, Supplementary Tables 1-2 and Supplementary References
