# Supplementary material for: Hyaluronan modulates TRPV1 channel opening, reducing peripheral nociceptor activity and pain
Source: Nat Commun. 2015 Aug 27;6:8095. doi: 10.1038/ncomms9095 (PMC4560824; doi:10.1038/ncomms9095)
Supplement: Supplementary Information — Supplementary Figures 1-7, Supplementary Tables 1-2 and Supplementary References [file ncomms9095-s1.pdf]

Supplementary Figure 1

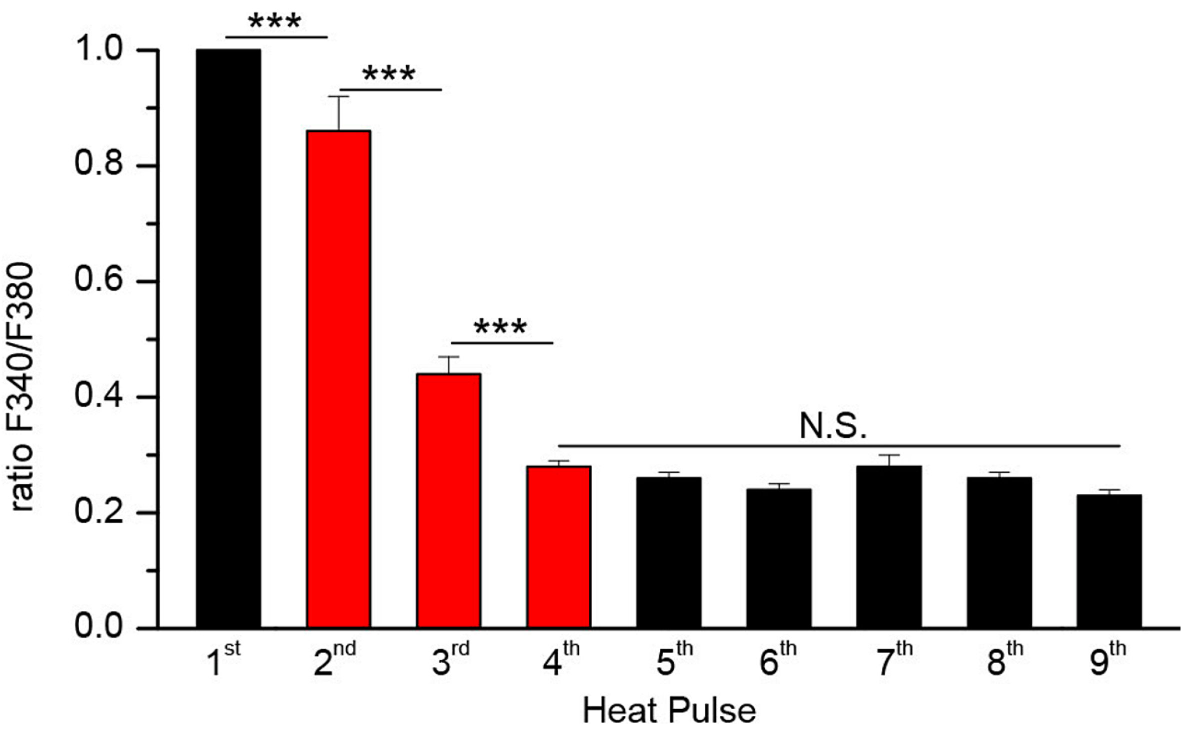

Supplementary Figure 1. **Washing does not reverse the inhibitory effect HA on intracellular calcium responses to heat in HEK-TRPV1-EYFP (+) cells.** The average amplitude change of responses to successive heat pulses (48 ºC) applied at 10 min intervals in cells (n=49) perfused with CS (black bars) and with a HA solution (red, bars). The maximal inhibitory effect was reached 30 min after the onset of perfusion with HA (4th pulse) and persisted after re-perfusion with CS. The data are represented as the mean ± s.e.m., Student's t-test ***P<0.001, $^{N.S}$ P>0.05.

Supplementary Figure 2

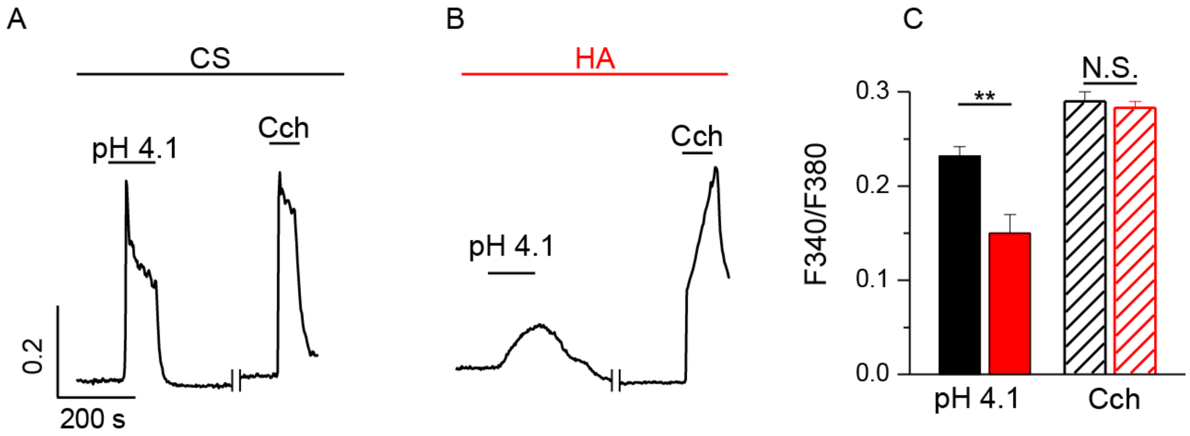

Supplementary Figure 2. Intracellular calcium change in a HEK-TRPV1-EYFP (+) cells in response to pH=4.1 .A, in cells perfused with CS and **B,** after exposure to HA initiated 30-60 min earlier. **C.** The average amplitude of the response to pH=4.1 (filled bars) and 100 μM Carbachol (Cch, striped bars, n=100) under perfusion with CS (black, n=74) and HA (red, n=10). The data are represented as the mean ± s.e.m., (Student's t-test $^{**}P<0.01$, $^{N.S.}\ P>0.05$

Supplementary Figure 3

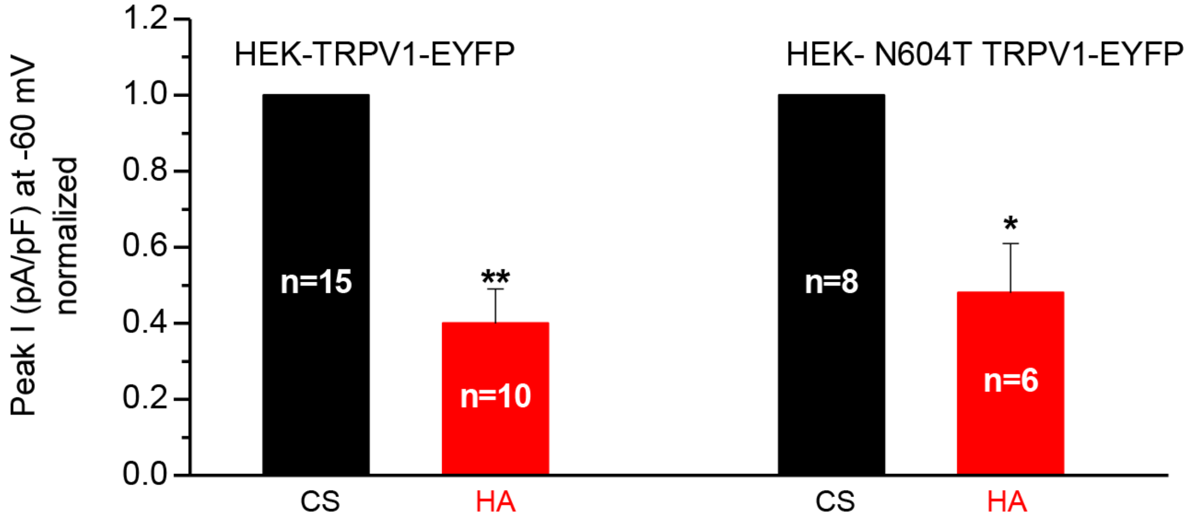

Supplementary Figure 3. Inhibitory effect of HA on HEK-N604T-TRPV1-EYFP (+) and TRPV1-EYFP HEK cells. The normalized peak amplitude of density current at -60 mV in response to 1 µM CAP in CS (black column) and in the presence of HA (red column). No differences in the response to CAP under HA were observed between both types of cells. The data are represented as the mean ± s.e.m. (Student's t-test: ** $P<0.01$, *$P<0.05$).

Supplementary Figure 4

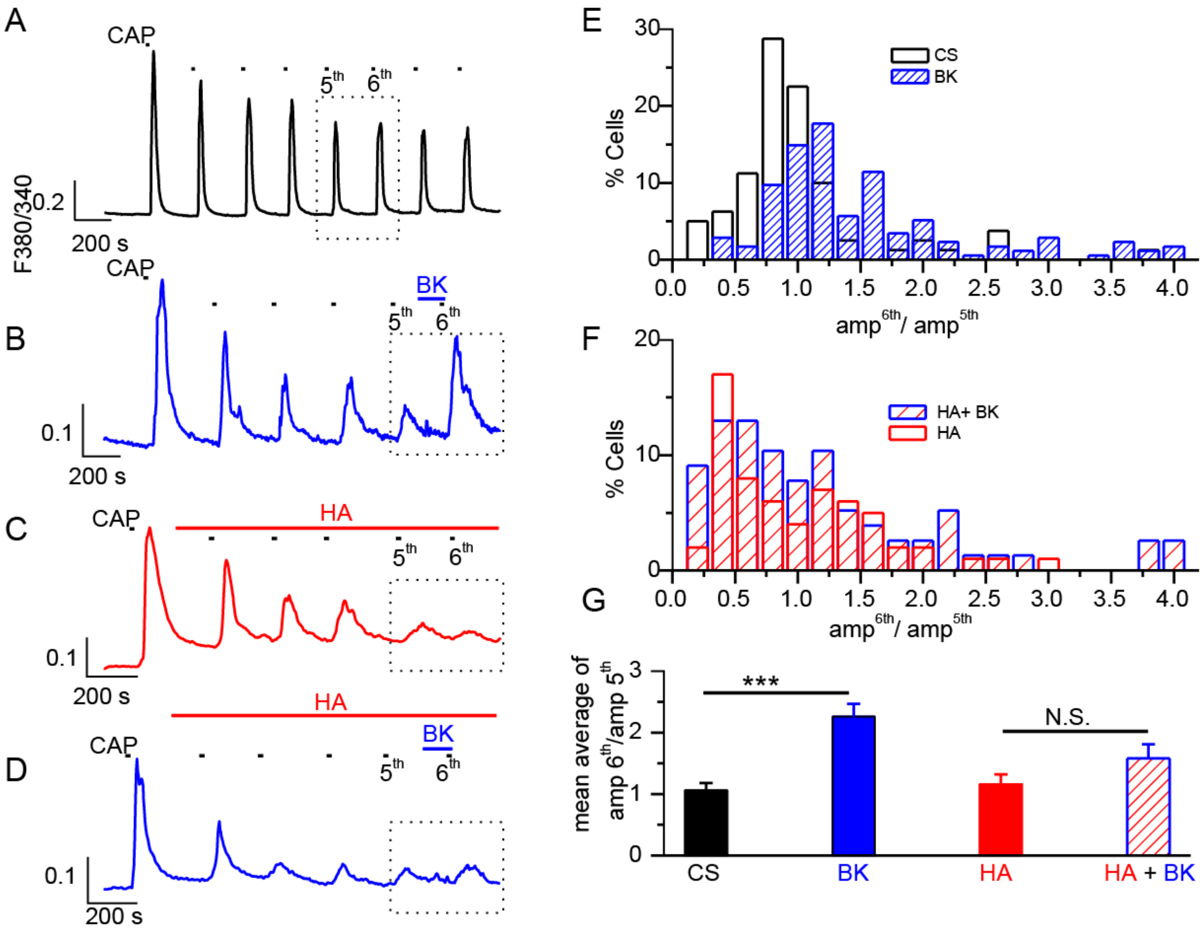

Supplementary Figure 4. Inhibition by HA of TRPV1 sensitization by BK. A-D. Examples of the $[Ca^{2+}]_i$ rises evoked by capsaicin in neonatal (P1-P4) DRG neurons. **A**. Recording performed under perfusion with CS (n=101). **B**. A similar experiment but with the application of 2 μM BK (n=170). **C**. The response to the same protocol as in A but under perfusion with HA. **D**. The response during exposure to HA and an application of 2 μM BK 2 min before the 6th stimuli to illustrate the absence of sensitization of the CAP response by BK. **E, F.** The frequency distribution of the ratio between the amplitude of the $[Ca^{2+}]_i$ responses to the 6th and 5th stimuli under control conditions (n=80) and when BK has been applied indicating a shift to the right of the ratio under BK (n=175), the effect under perfusion with CS (E) and the absence of this BK effect with exposure to HA (F). **G.** The mean value of the ratio of the responses to the 6th and 5th stimuli under HA (n=66) and under HA + BK (n=77). The data are represented as the mean ± s.e.m., Student's t-test: *** $P < 0.001$, $^{N.S.}$ $P > 0.05$.

Supplementary Figure 5.

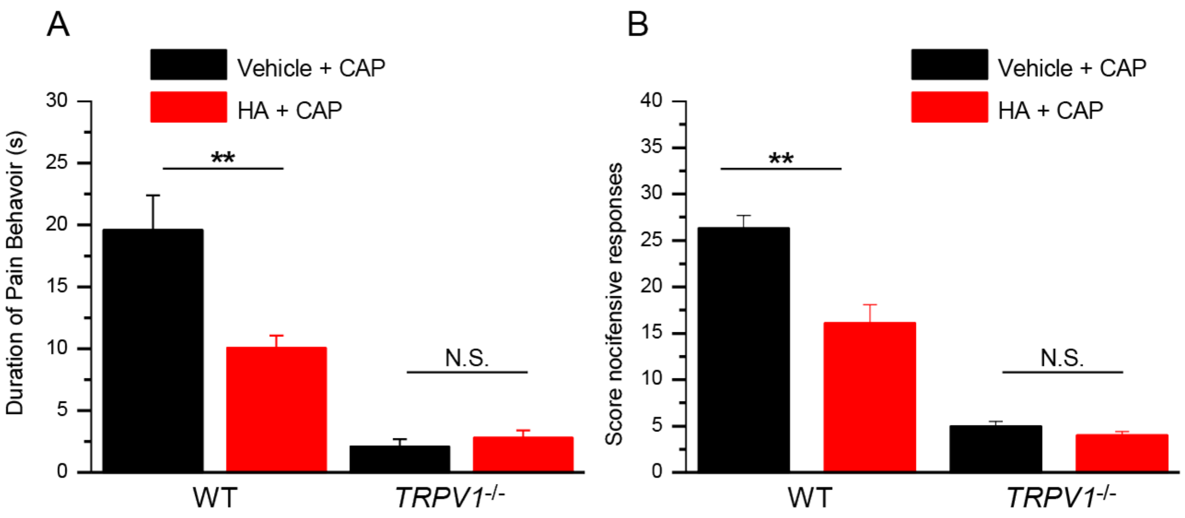

Supplementary Figure 5. Effect of HA on behavioral nocifensive responses to an intradermal CAP injection in the paw. The duration (**A**) and number of nocifensive behavior movements (licking, biting, lifting, guarding or shaking of the hind paw) (**B**), evoked by an intradermal CAP injection (10 µl of 1 µg/ml) that was preceded two days earlier by a 10-µl injection of either the vehicle or HA. Wild type (n=7) and *TRPV1* $^{-/-}$ (n=8). The data are represented as the mean ± s.e.m., Student's t-test: ** *P*< 0.01, $^{N.S.}$ *P* >0.05.

Supplementary Figure 6

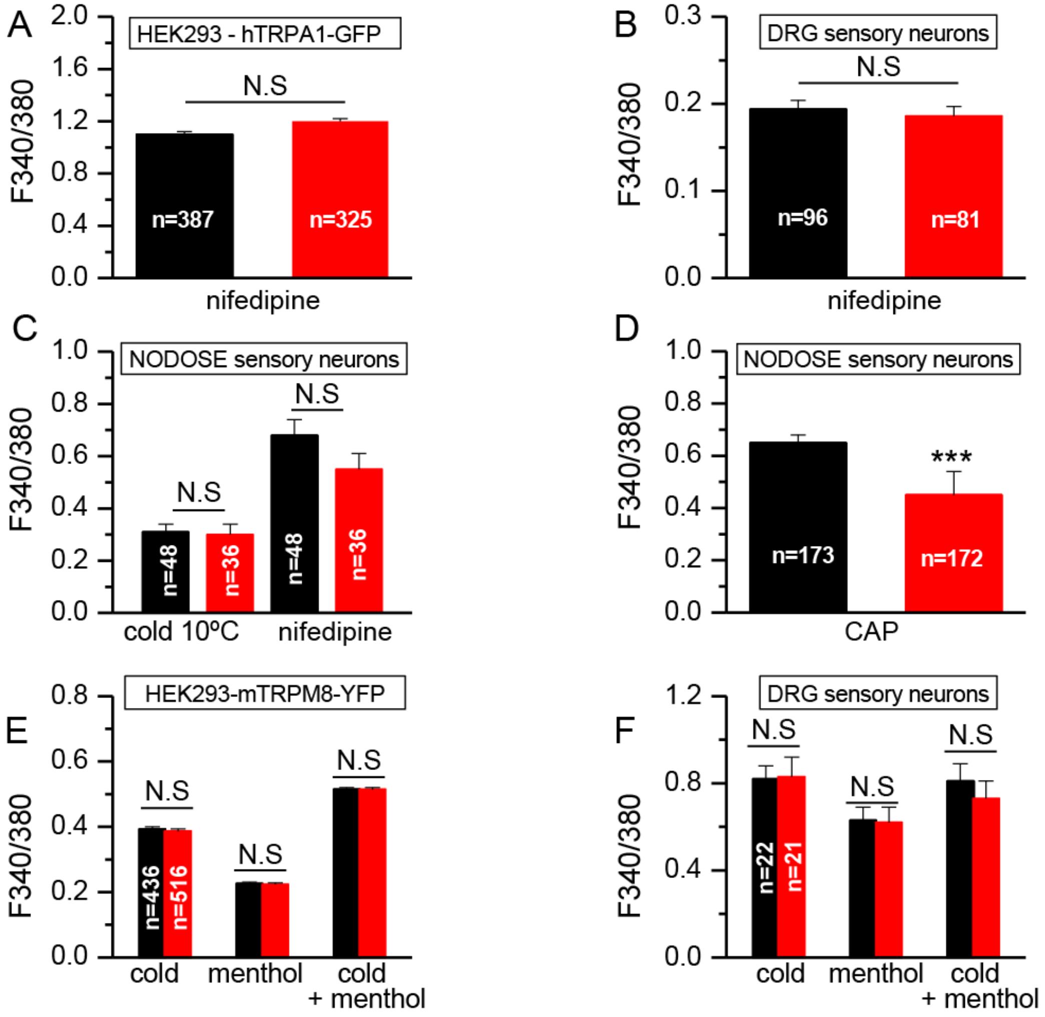

Supplementary Figure 6. The effects of HA on TRPA1 and TRPM8 channels. A-B. The average amplitude of the $[Ca^{2+}]_i$ response to 10 µM nifedipine in CS (black bars) and during perfusion with HA (red, bars) in HEK293-hTRPA1-GFP(+) cells and DRG cultured neurons, **C-D.** The average amplitude of the $[Ca^{2+}]_i$ response to cold (10 ºC) and 1 µM nifedipine in the same neurons (C) and 100 nM CAP (D) in cultured nodose ganglion neurons **E-F.** The average amplitude of $[Ca^{2+}]_i$ response in HEK293-mTRPM8-YFP cells and DRG cultured sensory neurons to cold, 100 µM menthol, and cold + 100 µM menthol, in CS (black bars) and during perfusion with HA (red, bars). Black bars: in external CS, Red bars: in HA solution. The data are represented as the mean ± s.e.m., Student's t-test: *** $P < 0.0001$, $^{N.S.}P > 0.05$.

Supplementary Figure 7

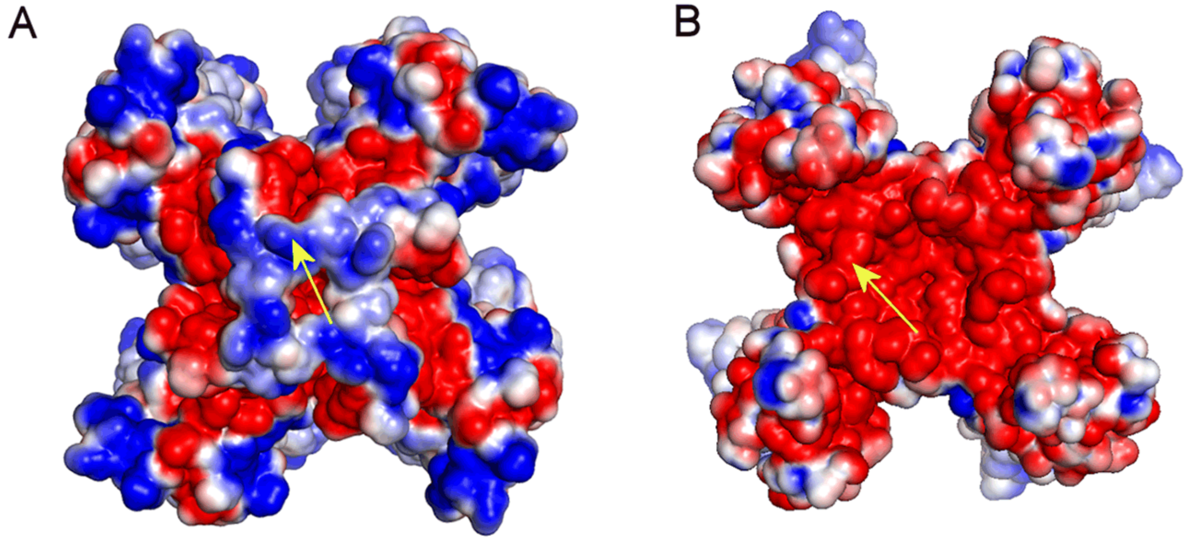

Supplementary Figure 7. Electrostatic potential of TRPV1 and TRPA1 models. Top view of the TRPV1 (**A**) and TRPA1 (**B**) models in electrostatic potential APBS representation[1] by using the plugin by M.G. Lerner and H.A. Carlson (2006) University of Michigan, Ann Arbor implemented in PyMol 1.6 (http://www.pymol.org). Positively charged regions are shown in blue and negatively charged domains in red. Arrows point to S5-pore helix loops.

Supplementary Table1

	**HEK-TRPV1-EYFP (+)**		**DRG sensory neurons *WT***		**DRG sensory neurons *$TRPV1^{-/-}$***	
	**CS n=65**	**HA n=42**	**CS n=49**	**HA n=45**	**CS n=135**	**HA n=98**
**Threshold ºC**	42±0	42±0	39.3±0.2	38.1±0.3	38.8±0.2	40.6±0.4
**Time to peak of** $[Ca^{2+}]_i$ **response (ms)**	38±6	24±3 *	44±1	27±3 *	28±4	24±5
**Amplitude of the response F340/F380**	0.37±0.02	0.26±0.02 ***	0.35±0.01	0.13±0.02 ***	0.39±0.01	0.31±0.01 **
**% inhibition**	30*		63*		26*	
**% of cells that respond**	93	91	41	28*	58	47*

Supplementary Table 1. Effect of HA on HEK-TRPV1-EYFP (+) cells and DRG sensory neurons from *$TRPV1^{-/-}$* mice. $[Ca^{2+}]_i$ changes were measured in HEK-TRPV1-EYFP (+) cells and cultured DRG primary sensory neurons from WT and *$TRPV1^{-/-}$* mice. We measured changes in $[Ca^{2+}]_i$ evoked by a brief heat stimulus (48 ºC) applied during perfusion with CS and in cells previously exposed to HA. A single heat stimulus was applied to prevent TRPV1 desensitization. No response to CAP was observed in cultured DRG sensory neurons of the *$TRPV1^{-/-}$* mice. (Student's t-test: *** P< 0.001,**P<0.01,*P<0.05).

Supplementary Table 2

	CS Average ratio F340/380	HA Average ratio F340/380	Change evoked by HA
CHO-mTRPA1cells Stimulus= CA 50µM	2.02 ± 0.05 n= 55	2.36 ± 0.05 n= 61***	Increment of 17%
DRG primary sensory neurons Stimulus= CA 50µM	0.35 ± 0.04 n= 32	0.31 ± 0.02 n= 38 $^{N.S}$	No effect
HEK hTRPA1-GFP cells Stimulus= MO 20µM	0.72 ± 0.02 n= 81	0.63 ± 0.02 n= 102**	Reduction of 13%
CHO-mTRPA1 cells Stimulus= MO 20µM	1.6 ± 0.1 n= 141	1.2 ± 0.1 n= 177 $^{N.S**}$	Reduction of 25%
HEK hTRPA1-GFP cells Stimulus= Nifedipine 10µM	1.1 ± 0.1 n= 325	1.2± 0.1 n= 331 $^{N.S}$	No effect
DRG primary sensory neurons Stimulus= Nifedipine 10µM	0.19 ± 0.01 n= 96	0.19 ± 0.01 n= 81	No effect

Supplementary Table 2. Effect of HA on TRPA1. $[Ca^{2+}]_i$ changes were measured in the CHO-mTRPA1 cell line in cultured DRG primary sensory neurons and HEK293 cells transfected with hTRPA1-GFP stimulated with cynnamaldehide (CA) or mustard oil (MO). Akin to other groups[1], we observed that the stimulation of TRPA1 with electrophilic compounds CA and MO yields highly variable results. More consistent activity was recorded when nifedipine or cold (35 to 10 ºC) were used as stimuli to activate TRPA1. The variability of the results is indicated in the last column. The data with nifedipine as a stimulus, in which no effect of HA was consistently observed are also included. (Student's t-test: *** $P<0.001$,**$P<0.01$, $^{N.S.}$ $P>0.05$).

Supplementary References

1. Baker, N.A., Sept, D., Joseph, S., Holst, M.J. & McCammon, J.A. Electrostatics of nanosystems: application to microtubules and the ribosome. *Proceedings of the National Academy of Sciences of the United States of America* **98**, 10037-10041 (2001).

2. Nilius, B., Appendino, G. & Owsianik, G. The transient receptor potential channel TRPA1: from gene to pathophysiology. *Pflugers Archiv : European journal of physiology* **464**, 425-458 (2012).
